# Cell-Mediated Proteomics, and Serological and Mucosal Humoral Immune Responses after Seasonal Influenza Immunization: Characterization of Serological Responders and Non-Responders

**DOI:** 10.3390/vaccines12030303

**Published:** 2024-03-14

**Authors:** Hanna Carlsson, Lars Brudin, Lena Serrander, Jorma Hinkula, Ivar Tjernberg

**Affiliations:** 1 Department of Clinical Chemistry and Transfusion Medicine, Region Kalmar County, 39244 Kalmar, Sweden; 2 Department of Biomedical and Clinical Sciences, Linköping University, 58183 Linköping, Sweden; 3 Department of Clinical Physiology, Region Kalmar County, 39244 Kalmar, Sweden; 4 Department of Medicine and Health Sciences, Linköping University, 58183 Linköping, Sweden; 5 Department of Clinical Microbiology, Region Östergötland, 58185 Linköping, Sweden; 6 Department of Molecular, Infection and Inflammation Centre, Linköping University, 58183 Linköping, Sweden

**Keywords:** immunization, influenza, vaccination, immune response, cellular immune response, humoral immune response, proximity extension assay, elderly

## Abstract

Immunization against influenza through vaccination is the most effective method with which to prevent infection. To assess protection after immunization, analysing humoral response with a hemagglutinin inhibition assay is the gold standard, but cell-mediated immune response has been shown to better correlate with protection in the elderly. Our aim was to explore the influenza-specific cell-mediated and mucosal humoral responses in serologically defined responders and non-responders. We analysed sera for total immunoglobulins (Ig) A, G, and M and nasal swab samples for influenza-specific IgA. Peripheral blood mononuclear cells were stimulated with trivalent influenza vaccine VaxiGripTetra, and supernatants were analysed for influenza-specific responses with the Olink Immune-Oncology panel using a proximity extension assay. We included 73 individuals, of which 69 completed the study with follow-up sampling at one and six months post-vaccination. Of the 73, 51 (70%) were found to be serological responders and 22 (30%) were non-responders. We did not find any significant differences in sex or mucosal humoral response between responders and non-responders; however, a higher IFNγ/IL-10 ratio in individuals ≤65 years of age indicates an enhanced cell-mediated immune response in this age group. Characteristics of the non-responders were found to be higher levels of IgM, Granzyme B and Interleukin 12, and lower levels of C-X-C motif chemokine 13 compared with those of the responders. In conclusion, our results did not show any correlation between serological response and age. Furthermore, the majority of influenza-specific cell-mediated immune markers did not differ between responders and non-responders; the immune marker profile of the non-responders and its contribution to protection is of interest but needs to be further explored.

## 1. Introduction

Influenza is a respiratory tract infection that affects the human health globally. Influenza is present seasonally and can cause pandemics. Influenza can give rise to a wide range of symptoms from asymptomatic to respiratory symptoms with or without the involvement of lungs and other internal organs. Infection also increases the risk of secondary bacterial pneumonia [1,2]. Because preventative measures such as good hand hygiene and social distance are rarely enough to prevent influenza from causing illness, vaccination against influenza is offered and is the most effective method to prevent infection [1,2]. Yearly influenza vaccination is offered to the population of Sweden, and the groups most in need of immunisation are, for example, children and adults with impaired lung and/or heart function [3], those who are immune-suppressed, the pregnant and the elderly [1,4]. Annually, the variation in the ability to mount adequate protection ranges from 28 to 87% [5,6,7]. Even though the mentioned groups seriously require the establishment of an adequate immune response, protection against influenza infection largely varies between individuals. Factors that affect the response are, for example, how well the virus strains included in the vaccine correspond to the circulating strains, health status, the individual immune response, and, for the elderly (>65 years of age), immunosenescence [4,8]. The haemagglutinin inhibition (HAI) assay is largely used to assess and evaluate serological response to influenza vaccines, and values of 1:40 or more are considered to correlate to 50% protection against infection in adults [9,10]. To classify an individual as a responder to the immunisation, the HAI antibody titre must show a four-fold rise or show results of seroconversion four to six weeks after vaccination compared to baseline [11,12].

Although humoral response to immunisation is the gold standard used to assess protection, cell-mediated immune response has been shown to be of great importance and needs to be further explored. Previous studies on cellular immune response after influenza immunisation include a wide range of immune markers representing different cell types and signalling pathways, for example, interferon (IFN)-γ and tumour necrosis factor (TNF), secreted by T helper (Th) 1; interleukin (IL)-4, IL-5, IL-9 IL-10 and IL-13, secreted by Th2-cells; IL-2 and IL-12, secreted by cytotoxic T lymphocytes (CTL); and granzyme B (GZMB), a marker of T-cell-mediated cytotoxicity [13,14,15,16]. In the elderly, cellular immune response has been shown to correspond to protection against influenza infection instead of serological response [15,16,17]. The immune system and immune response are complex processes. Traditionally, the immune response has been researched with methods that measures one immune marker at a time with, for example, the enzyme-linked immunosorbent assay (ELISA). New techniques displaying large-scale protein profiling with high sensitivity for a wide range of immune markers are now available on the market. One such technology is the proximity extension assay (PEA). The PEA technology enables the investigation of a wide range of proteins present in a small sample of, for example, plasma, sera, or supernatants from cell stimulation.

In this study, we aimed to investigate the immune responses of serologically defined responders and non-responders after influenza immunisation. More specifically, we wanted to characterise the influenza-specific cellular and mucosal humoral immune response in relation to sero-responder status, including investigating the possibility of compensatory mechanisms in non-responders.

## 2. Materials and Methods

### 2.1. Study Population and Vaccine

Patients and health care workers were invited to participate in this prospective study when they attended the 2017/2018 seasonal influenza vaccination in Kalmar, Sweden. The inclusion criteria included individuals ≥18 years old who were offered immunisation against seasonal influenza. Exclusion criteria were contraindications for vaccination such as individuals hypersensitive against any of the active substances, excipients included in the vaccine, or protein from egg or chicken, individuals with fever disease and individuals with impaired decision-making ability.

The vaccine used for vaccination in the 2017/2018 season was the trivalent VaxigripTetra (Sanofi AB, Stockholm, Sweden) vaccine. The influenza strains included in the vaccine were A/Michigan/45/2015 (H1N1)pdm09-like virus, A/Hong Kong/4801/2014 (H3N2)-like virus and B/Brisbane/60/2008-like virus.

At inclusion (pre-vaccination), blood and nasal swabs were collected together with a health inquiry for information on sex, age, length, weight, chronic diseases, lung disease, upper respiratory tract infections in the last five years and medication. Follow-up visits were scheduled four weeks and six months post-immunisation. At follow-up visits, blood and nasal swab were collected together with health inquiries for information on the side effects of the immunisation.

### 2.2. Total Serum Immunoglobulin A, G and M

Total IgA, IgG and IgM were measured in serum with immune-nephelometry on BN ProSpec Nephelometer (Siemens Healthcare Diagnostics, Tarrytown, NY, USA) in accordance with the manufacturer’s instructions.

### 2.3. Isolation of Peripheral Blood Mononuclear Cells

Whole blood was collected in BD Vacutainer^®^ CPT™ Cell Preparation Tubes with sodium citrate (Becton, Dickinson and Company, Franklin Lakes, NJ, USA) and handled in accordance with the manufacturer’s instructions. In short, samples were centrifuged within two hours after collection at room temperature for 20 min at 1500× *g*, acceleration (acc) at 4 and deceleration (dec) at 0 (Hettich Rotina 380R, Stockholm, Sweden). After centrifugation, tubes were inverted 10 times and stored at room temperature until further preparation.

Peripheral blood mononuclear cells (PBMCs) were collected by pooling the separated cell suspension in a 50 mL tube (VWR, Stockholm, Sweden). All PBMCs were collected within 24 h after centrifugation. Cells were washed by adding phosphate-buffered saline (PBS) without calcium and magnesium (Gibco, Invitrogen, Paisley, Scotland, UK)). The cell suspension was then centrifuged for 12 min at 350× *g* (Hettich Rotina 380R, Stockholm, Sweden). The supernatant was discarded, and the cell pellet was resuspended. PBS was added and centrifuged for 12 min at 350× *g* (Hettich Rotina 380R, Stockholm, Sweden). The supernatant was discarded, and the washed cells were resuspended in foetal bovine serum (FBS) (Sigma-Aldrich, Taufkirchen, Germany) with 20% dimethyl sulfoxide (DMSO) (Sigma-Aldrich, Taufkirchen, Germany). The cell suspension was transferred to cryovials, placed in a cool cell at −80 °C for two hours and then transferred to a liquid nitrogen tank for cryopreservation until further preparation.

Prior to stimulation, the PBMC cryovials were transferred from liquid nitrogen to dry ice for transport. The cryovials were thawed in a 37 °C water bath for ten minutes. After thawing, the cells were transferred to a sterile 15 mL Falcon tube (VWR, Stockholm, Sweden). The cryovials were washed with warm (37 °C) AIM V medium (Gibco, Invitrogen, Paisley, Scotland, UK) to ensure that the maximum number of cells was transferred. Warm AIM V was added, PBMCs were washed via centrifugation at 400× *g* for five minutes (Hettich Rotina 380R, Stockholm, Sweden) and the supernatant was discarded. The PBMCs were resuspended in warm AIM V and then centrifuged at 400× *g* for five minutes (Hettich Rotina 380R, Stockholm, Sweden). The supernatant was discarded, and the PBMCs were resuspended in warm AIM V. Cells were counted in a Bürker chamber. Dead cells were stained with Trypan blue. The final concentration was adjusted to 1 × 10^6^ live PBMC/450 µL.

### 2.4. Stimulation of Peripheral Blood Mononuclear Cells with Influenza Vaccine

PBMCs (1 × 10^6^ cells) were stimulated with the trivalent influenza vaccine VaxigripTetra, containing virus strains A/Michigan/45/2015 (H1N1)pdm09-like virus, A/Hong Kong/4801/2014 (H3N2)-like virus and B/Brisbane/60/2008-like virus (2017/18, Sanofi AB, Stockholm, Sweden) at a final concentration of 0.5 µg/mL in a 48-well plate (Nunc A/S, Roskilde, Denmark) for 20 h at 37 °C with 5% CO_2_ and 95% relative humidity. Concanavalin A (Sigma-Aldrich, Stockholm, Sweden) was used as a positive-culture control at a final concentration of 2 µg/mL, and AIM V was used to for the culture of control cells. The procedure for stimulation by Gijzen et al. [14] was applied with the following modifications: we used 1 × 10^6^ cells, influenza vaccine as a stimulant (the concentration was determined in pilot studies) and only a medium as a negative or background control. After stimulation, the supernatants were collected and stored at −80 °C until further analysis.

To investigate the effect of using cryopreserved PBMCs in our stimulations, we also performed the stimulation of freshly collected PBMCs. From a separate single donor not included in the study population, PBMCs were collected and cryopreserved at the start of the study, and at the end of the stimulations, fresh PBMCs were collected from the same donor. The cryopreserved and fresh cells were stimulated simultaneously in the same way as those from the study participants were, and the supernatants were collected and stored at −80 °C until further analysis.

### 2.5. Haemagglutination Inhibition Test, HAI

Influenza-specific antibody titres were measured with a haemagglutination inhibition test in accordance with the WHO manual for the laboratory diagnosis and virological surveillance of influenza [18]. Influenza virus strains used in the HAI test were A/H1N1/09pdm, A/H3N2/HK and B/60/Brisbane2008 (Sino Biological, Eschborn, Germany). The virus strains were selected to correspond to the strains included in the vaccine. Responders were defined as those showing a four-fold rise in antibody titre or seroconversion against one or more virus strains at the one month follow-up [12].

### 2.6. Influenza-Specific Mucosal Immunoglobulin A (IgA)

Nasal swab samples were analysed for influenza-specific IgA with an ELISA as previously described [19,20]. Antigens used in the analysis were A/H1N1/Brisbane/02/2018, A/H3N2/Kansas/2018 and B/Brisbane/60/2008-like (Sino Biological, Eschborn, Germany), and correspond to the strains included in the vaccine.

### 2.7. Proximity Extension Assay

An Immuno-Oncology panel (Olink Proteomics, Uppsala, Sweden) was used to analyse supernatants from influenza vaccine-stimulated PBMCs with PEA technology. The Immuno-Oncology panel includes the measurement of 92 proteins involved in immune response and immuno-oncology diseases, and the processes included are, for example, chemotaxis, apoptosis and cell killing, tumour immunity, vascular and tissue remodelling, and also metabolism and autophagy [21]. The choice of this specific panel was based on the inclusion of previously researched biomarkers shown to be involved in the immune response after influenza immunisation [13,14,15,16]. Samples were distributed randomly in 96-well plates and shipped frozen to Clinical Biomarkers Facility, SciLifeLab, Uppsala University, and analysed accordance with the manufacturer’s recommendations. In brief, our supernatant samples were incubated with 92 pairs of antigen-specific antibodies with attached oligonucleotides (PEA probes). Under incubation, the antibody pairs bind to their antigen, the probes comes close enough to hybridize, the addition of DNA polymerase promotes extension, and DNA templates are formed. The DNA templates are then amplified, unbound primers are removed, and detection and quantification are carried out via a microfluidic quantitative polymerase chain reaction [22]. Protein concentrations are presented in a relative quantification unit on a log2 scale named normalized protein expression (NPX). Because NPX values are not an absolute concentration, calculations of ratios between proteins are difficult.

### 2.8. Statistical Analysis

The continuous variables are presented as median and quartiles, and the categorical variables are presented as numbers (frequencies) for all groups; appropriate differences are analysed using Mann–Whitney’s U-test (MW-U-test) for continuous variables, and Fisher’s exact test is used for categorical variables (Tables 2 and 3).

**Figure 1:**
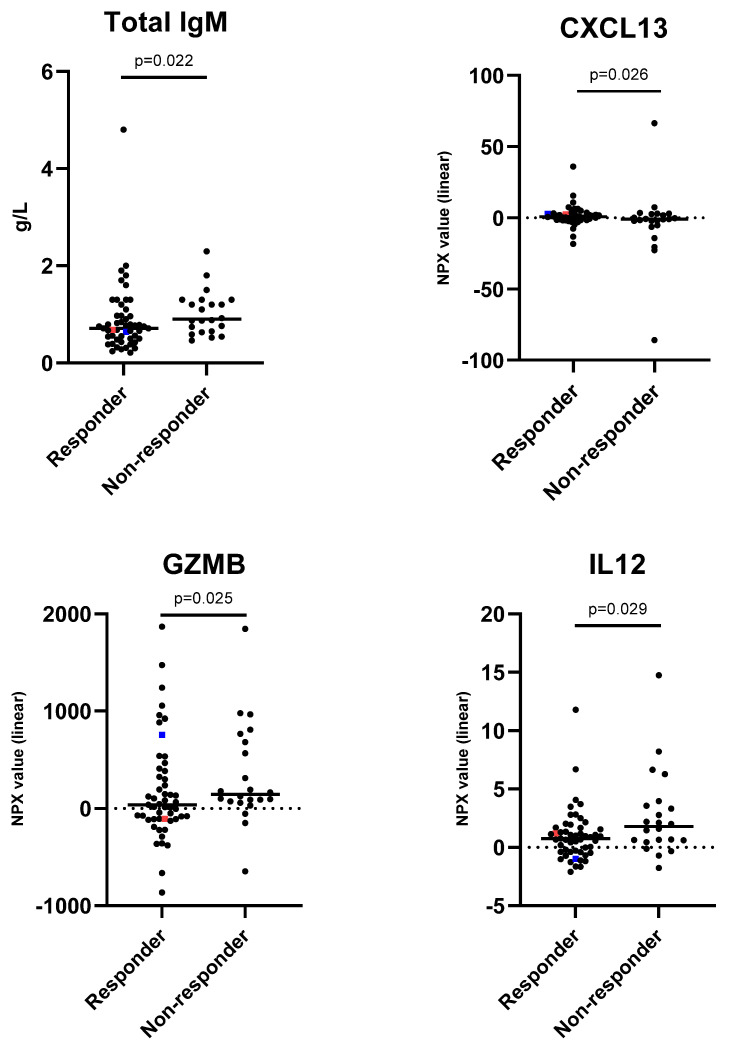
Protein characteristics of responders and non-responders at one-month post immunisation. Data are presented as g/L for total IgM and normalized protein expression (NPX) values are presented on a linear scale for C-X-C motif chemokine (CXCL13), Granzyme b (GZMB), and interleukin 12 (IL12). The dotted bar is set at the median, and *p*-values are from the multivariate logistic regression. Red squares show values of participants with immunosuppressive medication, and blue squares show values of participants with a vaccine breakthrough. Black dots represent the remaining study participants.

All proteins were initially expressed on a 2logaritmic scale and converted into normalized protein expression (NPX) values on a linear scale (median and quartiles of all proteins are shown in Supplementary Table S1 for the three occasions) to allow for background subtraction. Group differences using the MW-U-test at one month with *p* < 0.1 were then qualified for the univariate and multivariate logistic regressions as follows: The continuous variables were transformed to their quartiles (except sex and age categories; see Table 4), and these were then used as continuous variables in the model. This was carried out to minimize the number of statistical analyses, to avoid outliers and to be able to judge if the trend was interrupted at some quartile. Statistica version 13.5.0.17 (TIBCO Software, Inc., Palo Alto, CA, USA) was used for all statistics, and *p*-values < 0.05 were considered statistically significant. GraphPad Prism version 8 (GraphPad Software Inc., La Jolla, CA, USA) was used for Figure 1.

## 3. Results

### 3.1. Study Population

In total, 73 individuals (71% female and 29% male) were included in the study, of which 69 completed it with follow-up samples. Altogether, 51 (70%) individuals were found to be responders to influenza immunisation and 22 (30%) were found to be non-responders, according to the HAI test at the one-month follow-up. Of the 51 responders, 29 (57%) individuals remained responders at the six-month follow-up. The HAI titre results for the ingoing influenza strains according to responder and non-responder status are shown for the one-month and the six-month follow-up in Table 1. The responders at one month consisted of 37 females and 14 males, with a median age of 77 years, and those at six months consisted of 19 females and 10 males, with a median age of 77 years. One individual reported immunosuppressive medication, and 10% experienced side effects after the immunisation.

The non-responders consisted of 15 females and 7 males, with a median age of 73 years. No individuals reported immunosuppressive medication, and 14% reported side effects from the immunisation. The side effects reported for both groups were general malaise, headache, muscle and/or joint ache and fever. Of the 22 non-responders at one-month, 2 were found to be responders at the six-month follow-up. Characteristics of responders and non-responders are presented in Table 2. No significant differences were found between responders and non-responders for any of the characteristics.

### 3.2. Total Serum Immunoglobulins and Specific Mucosal Immunoglobulin A

Total serum immunoglobulin A, G and M were measured via immune nephelometry, and no significant differences were found for IgA and IgG in relation to responder status. For IgM, a *p*-value of 0.053 was observed, with a median of 0.7 g/L for responders and 0.9 g/L for non-responders (Table 3).

The presence of influenza-specific mucosal IgA was investigated with an ELISA. No significant differences were found in the levels of specific mucosal IgA for either researched antigens or at any time point between responders and non-responders. Although both responders and non-responders had an increase in specific mucosal IgA at the one-month follow up for all researched antigens with a ratio >1, no significant differences between groups were found (Table 3).

### 3.3. Protein Profiling of Supernatants from PBMCs Stimulated with Influenza Vaccine VaxigripTetra

Supernatants from influenza vaccine-stimulated PBMCs were analysed with the Olink Immuno-Oncology panel. A univariate logistic regression of the 92 proteins included in the panel showed that three proteins, C-C motif chemokine (CCL19) (*p* = 0.048), IL-13 (*p* = 0.042) and GZMB (*p* = 0.046), significantly differed between responders and non-responders and at the one-month post-immunisation time mark (Table 4). No significant differences were found between our groups either at inclusion or six months post-immunisation for any of the 92 proteins (Table S1).

### 3.4. Characterisation of Influenza Immunisation Responders and Non-Responders

To further characterise potential differences between responders and non-responders, a multivariate logistic regression model was used. Protein biomarkers with a *p*-value <0.1 in the univariate statistical analysis were included in the multivariate model together with total IgM, sex and age. Thus, the included markers in our regression were sex, age, total IgM, CCL19, CD70, C-X-C motif chemokine (CXCL) 13, IL13, GZMB and IL12. We found that after correction for sex and age, the immune characteristics of the responders after influenza immunisation were lower concentrations of IgM, GZMB and IL12, and a higher concentration of CXCL13 (Figure 1), in comparison with those of the non-responders.

### 3.5. Immunosuppressive Medication

One female participant reported using immunosuppressive medication: methotrexate. The participant was 82 years of age. She was found to be a responder in the HAI test. She was prescribed methotrexate for the treatment of rheumatoid arthritis, and she reported no side effects from the immunisation aside from mild myalgia/arthralgia. More characteristics of this patient may be found in Table S2 and in Figure 1.

### 3.6. Vaccine Breakthrough

One study participant was found to have a vaccine breakthrough infection. The participant was a man 78 years of age. He was found to be a responder in the HAI test. He reported no chronic disease, was on treatment with apixaban and atorvastatin, and had no reported side effects from the immunisation. In February 2018, he sought medical care and was tested for influenza. He was found to be negative for influenza A and positive for influenza B/Yamagata. Protein characteristics of the participant are shown in Table S2 and Figure 1.

### 3.7. Age-Associated Immune Response

Cell-mediated immune response represented by the ratio of IFN γ/IL-10 between the age group of ≤65 years and that of >65 years showed a significant difference in expression, with *p* < 0.0001, at the one-month follow-up, but not at inclusion or the six-month follow-up. The age group of ≤65 years displayed a median ratio of 72 (range: −240–133), and that of >65 years displayed a median expression of 9 (range: −791–2815). No significant differences were observed for the ratio of Grz B/IL-10 at any sampling time between the age groups.

### 3.8. Comparison of PBMC Stimulations Using Fresh and Cryopreserved Cells

To investigate how the PBMCs’ cell-mediated immune response was affected by cryopreservation, stimulation was performed with fresh as well as cryopreserved cells collected from a separate single donor not participating in the study. Cryopreserved and fresh cells were stimulated simultaneously. The supernatants were analysed with the Olink Immuno-Oncology panel. We then compared influenza-specific NPX-values for the protein markers included in our multivariate logistic regression and found that freshly stimulated cells and cryopreserved cells displayed values in the same quartile for CXCL13 (fresh > 1.1 and frozen > 1.1) and IL12 (fresh > 1.2 and frozen > 1.2). For GZMB, freshly stimulated cells displayed one quartile lower concentration (15–259) than the cryopreserved cells did (>259). Quartiles for CXCL13, IL12 and GZMB are displayed in Table 4.

## 4. Discussion

In the current prospective study, we explored the humoral, mucosal and cellular immune response after immunisation with the trivalent influenza vaccine VaxigripTetra, which was used for the vaccination of the general public of Sweden in the season 2017/2018. We measured influenza-specific antibodies with HAI and divided our study population into two groups according to serological responder status one month post-immunisation. We found that 70% were responders to the immunisation, which corresponds well with what has been reported to the European I-MOVE (Influenza- Monitoring Vaccine Effectiveness) network [23]. No significant differences were found for either age groups or sex when comparing responders with non-responders. A possible explanation for the absence of an age-dependent reduction in serological response in our study population could be due to a selection bias towards the reasonably healthy elderly. Inclusion in our study required the individuals to independently visit the health care centre for vaccination, thus excluding elderly individuals residing in nursing homes. Of the serological responders at one month, 57% remained responders at the six-month follow-up. We also found two non-responders to be serological responders at six months, which might have been due to influenza virus exposure in the period between the one-month and six-month follow-up and/or a delayed humoral response to the immunisation.

The season 2017/2018 was unusual in Sweden, with influenza B/Yamagata strain dominating most of the season [23]. The trivalent vaccine VaxigripTetra included two influenza A strains (A/Michigan/45/2015(H1N1)pdm09-like virus and A/HongKong/4801/2014(H3N2)-like virus) and one influenza B strain (B/Brisbane/60/2008-like virus), but lacked an influenza B/Yamagata strain. The absence of an influenza B/Yamagata strain in the vaccine was the probable cause of the vaccine breakthrough for one of the participants. Otherwise, none of the participants sought medical care for influenza illness regardless of responder status. To overcome the possible problem with vaccine breakthrough, it would be favourable to use a quadrivalent vaccine including two influenza A strains and two influenza B strains.

The influenza-specific nasal IgA response after immunisation did not significantly differ between responders and non-responders for any of in the vaccine included influenza strains. This is most likely due to the manner of administration of the vaccine. Although we did not observe any differences between the groups, both responders and non-responders showed an increase in influenza-specific nasal IgA one month post-vaccination. Therefore, even if the vaccine is intramuscularly or subcutaneously administrated, the main mediation of immune response ought to be through the skin and lymphatic system, but also appears to be upregulated in the nasal mucosal tissue.

Previous studies on humoral response to influenza infection have shown that total IgM might be a marker for the severity of infection [24,25]. Higher levels of total IgM present in serum or plasma have been associated with less severe infection. The non-responders displayed a higher level of total IgM in serum than the responders did. It could be speculated that this might have a compensatory effect for non-responders with higher levels of total IgM and lower levels of influenza-specific IgG antibodies.

In our study, we carried out a broader attempt to explore the cell-mediated immune response after immunisation. PBMCs were stimulated with VaxigripTetra, and supernatants were analysed for influenza-specific cellular immune response with PEA. Even though we analysed a large amount (92 proteins) of cellular immune markers representing a wide spectrum of immune cells and signalling pathways, the majority of cell-mediated immunity markers did not differ significantly between responders and non-responders. Only CXCL13, GZMB and IL12 together with total IgM seem to be linked to the serological response to immunisation.

CXCL13 is a chemokine acting as a chemoattractant for B-cells and is expressed by follicular T-cells [26,27]. Responders showed a higher expression of CXCL13 compared with non-responders and might be contributing to influenza-specific antibody production as characterized by this group.

GZMB is a serine protease found in granules of CTLs, and its main function is to initiate apoptosis [28,29]. The non-responders expressed higher levels of GZMB than did the responders. In previous studies, increased GZMB levels have been shown to correlate with protection against influenza after immunisation, especially in the elderly [15,16]. Even though we did not compare individuals with laboratory-diagnosed influenza illness in our study, one could speculate that the higher level of GZMB in the non-responders might contribute to protection in the absence of influenza-specific antibodies.

IL12 is a proinflammatory cytokine that contributes to the development of T_H_1 and T_H_17 cells, and is produced by dendritic cells, macrophages and B cells [30]. Besides promoting the development of CD4+ T-cells into T_H_1 cells, IL12 also influences CTL cytotoxicity and proliferation [31]. The responders displayed lower levels of both GZMB and IL12 compared with the non-responders, which may be an indication that the non-responders had an enhanced activation of CTL.

Thus, the serological non-responders after influenza immunisation seemed to be characterized by cell-mediated immunity with higher levels of total IgM, GZMB and IL12, and lower levels of CXCL13 than the responders. Because GZMB and IL12 secretions were increased in the non-responders, we can only speculate that cell-mediated immunity with the enhanced involvement of CTL might have compensated and contributed to protection in those individuals, but there might also be other mechanisms involved.

We did not observe any differences in cell-mediated immunity six months after the immunisation between serological responders and non-responders. This indicates that immune response following influenza immunisation is relatively short-lived, and it is of great importance to match the immunisation time with the influenza season.

Traditionally, research on immune responses after influenza immunisation and its association with protection includes serological response and, in some studies, cell-mediated immune responses. Cell mediated responses are, in previous studies, often represented by a few selected biomarkers. Because the immune system is very complex and includes a large network of cells and biomarkers, we wanted to explore immune responses to influenza immunisation through a wider perspective, including serological, cell mediated and mucosal humoral immune responses in parallel. The strengths of our study are the well-timed sampling and that we included a large variety of variables representing a broader perspective of immune responses to influenza immunisation. We observed a significant difference between the age groups of ≤65 years and >65 years for the ratio of IFNγ/IL-10. The age group of ≤65 years showed a higher ratio, indicating a higher cell-mediated immune response. These results are in line with those of previously presented research [15,16,17] and might contribute to protection against influenza infection after immunisation. The choice of using frozen PBMCs also did not seem to have a significant effect on the influenza-specific cell-mediated immune response results.

Although we have strengths in our study, there are also some limitations. First and foremost, our study population was limited due to practical reasons. We performed the study in an exploratory and hypothesis-generating manner above all for investigating cell-mediated immune responses with PEA technology. Since the results of our chosen panel are presented as NPX values instead of absolute concentrations, they only allow us to perform comparisons between our study groups and not between other studies and ours. To be able to draw further conclusions, more studies are needed and should include, for instance, a larger study population, repeated analysis, flow cytometry and the quantification of biomarkers of interest. We also acknowledge that the study participants representing both responders and non-responders may have had pre-existing HAI antibody titres of relevant levels indicating influenza protection. However, the present responder definition used in our study still indicates a major difference in serological response patterns from non-responders after recent immunisation. This difference is intriguing and encourages studies on alternative immune responses including cell-mediated responses.

## 5. Conclusions

In the present study, we found 70% of the participants to be serological responders after immunisation, and immunisation response did not depend on sex, but cell-mediated immune response seemed higher in the younger age group. Although the majority of cell-mediated immune markers did not differ between responders and non-responders, the cell-mediated immunity of the serological non-responders was characterised by higher levels of total IgM, GZMB and IL12, and lower level of CXCL13 compared with those of the responders. The involvement of these cell-mediated immune markers and their mechanisms contributing to protection against influenza infection in serological non-responders need to be further explored.

## Figures and Tables

**Table 1:** Summary of haemagglutinin inhibition titre assay results.

	Responders		Non-Responders	
Influenza Strain, Median (Range)	One Month (*n* = 51)	Six Months (*n* = 29)	One Month (*n* = 22)	Six Months (*n* = 44)
A/H1N1/09pdm	160 (5–20,480)	120 (5–10,240)	160 (60–5120)	160 (20–1280)
A/H3N2/HK	80 (5–10,240)	60 (5–5120)	80 (5–5120)	80 (5–2560)
B/60/Brisbane2008	160 (5–5120)	100 (5–2560)	320 (40–1280)	160 (20–640)

*n* = number of.

**Table 2:** Health inquiry.

Variable	Responders	Non-Responders	*p*-Value	Total
*n* (%)	51 (70)	22 (30)		73 (100)
Sex *n* (%)				
Female	37 (73)	15 (68)		52 (71)
Male	14 (27)	7 (32)	0.781	21 (29)
Age (yrs)				
Mean (SD)	70 (18)	67 (15)		69 (17)
Median (Q1–Q3)	77 (61–84)	73 (61–81)	0.243	76 (61–81)
Age categories n (%)				
≤65	14 (27)	9 (41)		23 (32)
>65	37 (73)	13 (59)	0.282	50 (68)
Height (cm)				
Mean (SD)	168 (9)	170 (7)		168 (9)
Median (Q1–Q3)	167 (160–175)	170 (164–175)	0.367	169 (161–175)
Weight (kg)				
Mean (SD)	76 (15)	77 (18)		76 (16)
Median (Q1–Q3)	75 (66–86)	75 (67–87)	0.967	75 (66–86)
BMI (kg m^−2^)				
Mean (SD)	27 (4)	27 (6)		27 (5)
Median (Q1–Q3)	27 (24–29)	25 (24–30)	0.539	27 (24–29)
Diabetes				
No	43 (84)	16 (76)		59 (82)
Yes	8 (16)	5 (24)	0.504	13 (18)
Lung disease				
No	45 (88)	20 (95)		65 (90)
Yes	5 (10)	1 (5)		6 (8)
Missing	1 (2)	0 (0)	0.662	1 (1)
Upper respiratory tract infections in last five years				
0–3	45 (88)	18 (86)		63 (88)
4–6	5 (10)	1 (5)		6 (8)
>6	0 (0)	1 (5)		1 (1)
Missing	1 (2)	1 (5)	0.233	2 (3)
Immune-suppressive medication				
No	50 (98)	21 (100)		71 (99)
Yes	1 (2)	0 (0)	>0.99	1 (1)
Side effects				
No	46 (90)	19 (86)		65 (89)
Yes	5 (10)	3 (14)	0.691	8 (11)

Differences between responders and non-responders analysed using Mann–Whitney U-test for continuous variables and Fishers exact test for categorical variables. *n* = number of; yrs = years; SD = Standard deviation; Q = Quartile; cm = centimetres; kg = kilogram; BMI = body mass index; kg m^−2^ = kilograms per square meter.

**Table 3:** Total serum immunoglobulins and influenza-specific mucosal immunoglobulin A at one month.

	ONE MONTH			
Variable	Responders	Non-Responders	*p*-Value	Total
*n*	51	22		73
Total serum IgA (g/L)				
Mean (SD)	2.56 (1.09)	2.54 (1.01)		2.55 (1.06)
Median (Q1–Q3)	2.5 (1.6–3.3)	2.7 (1.9–2.9)	0.891	2.6 (1.9–3.0)
Total serum IgG (g/L)				
Mean (SD)	10.7 (2.7)	11.1 (2.0)		10.8 (2.5)
Median (Q1–Q3)	11 (9–13)	11 (10–12)	0.587	11 (10–12)
Total serum IgM (g/L)				
Mean (SD)	0.88 (0.71)	1.03 (0.45)		0.93 (0.64)
Median (Q1–Q3)	0.7 (0.5–1.0)	0.9 (0.7–1.3)	0.053	0.8 (0.6–1.2)
Mucosal IgA A/Brisbane				
Mean (SD)	22.1 (34.1)	17.7 (17.3)		20.7 (29.6)
Median (Q1–Q3)	12.8 (7.0–21.3)	11.5 (6.6–22.2)	0.994	12.6 (7.0–22.0)
Mucosal IgA A/Kansas				
Mean (SD)	22.6 (27.8)	19.0 (17.1)		21.4 (24.7)
Median (Q1–Q3)	14.5 (8.3–22.9)	11.6 (8.7–22.2)	0.865	13.8 (8.5–22.6)
Mucosal IgA B/Brisbane-like				
Mean (SD)	20.4 (18.5)	21.0 (19.8)		20.6 (18.8)
Median (Q1–Q3)	15.0 (8.0–25.7)	12.6 (8.2–32.2)	0.960	14.2 (8.2–27.6)
Ratio Mucosal IgA A/Brisbane				
Mean (SD)	1.81 (1.94)	1.69 (0.89)		1.77 (1.67)
Median (Q1–Q3)	1.2 (0.8–2.2)	1.7 (0.9–2.5)	0.365	1.2 (0.8–2.3)
Ratio Mucosal IgA A/Kansas				
Mean (SD)	3.21 (9.44)	2.10 (1.42)		2.84 (7.71)
Median (Q1–Q3)	1.4 (0.9–2.0)	1.6 (1.1–2.8)	0.408	1.4 (0.9–2.4)
Ratio Mucosal IgA B/Brisbane-like				
Mean (SD)	2.96 (5.15)	2.03 (1.61)		2.66 (4.35)
Median (Q1–Q3)	1.3 (0.9–2.2)	1.6 (0.9–2.7)	0.720	1.4 (0.9–2.3)

Differences analysed using Mann–Whitney U-test for continuous variables and Fisher’s exact test for categorical variables. Differences at *p* < 0.1 are in bold. Ratios were calculated by dividing the one-month secretion with the baseline. *n* = number of; IgA = immunoglobulin A; g/L = grams per Litre; SD = Standard deviation; Q = Quartile.

**Table 4:** Characteristics of serological responders after influenza immunisation using univariate and multivariate logistic regression. Laboratory parameters are divided into quartiles.

		Responders		Univariate Logistic Regression		Multivariate Logistic Regression	
Parameter	Total	*n*	(%)	OR (95% Conf Int)	*p*-Value	OR (95% Conf Int)	*p*-Value
Sex							
Female	52	37	71	1.00		1.00	
Male	21	14	67	0.81 (0.27–2.45)	0.707	0.94 (0.22–4.03)	0.936
Age categories n (%)							
≤65	23	14	61	1.00		1.00	
>65	50	37	74	1.83 (0.63–5.33)	0.263	0.37 (0.07–2.04)	0.249
Total IgM (g/L)							
≤0.5	15	14	93	1.00		1.00	
0.6–0.8	26	19	73	0.54 (0.33–0.90)		0.49 (0.27–0.90)	
0.9–1.1	12	7	58	0.29 (0.11–0.80)		0.24 (0.07–0.81)	
>1.1	20	11	55	0.16 (0.04–0.72)	0.017	0.12 (0.02–0.72)	0.022
CCL19							
≤−1.1	19	13	68	1.00			
−1.2–0.6	18	18	100	0.62 (0.38–1.00)			
0.7–2.0	17	11	65	0.38 (0.15–0.99)			
>2.0	19	9	47	0.24 (0.06–0.99)	0.048		-
CD70							
≤−0.3	18	13	72	1.00			
−0.4–0.2	19	17	89	0.70 (0.43–1.13)			
0.3–0.9	19	11	58	0.49 (0.19–1.28)			
>0.9	17	10	59	0.34 (0.08–1.45)	0.143		-
CXCL13							
≤−1.7	18	9	50	1.00		1.00	
−1.8–−0.4	18	12	67	1.48 (0.92–2.38)		2.06 (1.09–3.88)	
−0.5–1.1	18	17	94	2.20 (0.86–5.66)		4.24 (1.20–15.02)	
>1.1	19	13	68	3.26 (0.79–13.46)	0.100	8.73 (1.31–58.19)	0.026
IL13							
≤−0.9	19	18	95	1.00			
−0.8–−0.06	17	10	59	0.61 (0.38–0.98)			
−0.05–0.7	17	11	65	0.37 (0.14–0.96)			
>0.8	20	12	60	0.23 (0.05–0.95)	0.042		-
GZMB							
≤−135	19	17	89	1.00		1.00	
−134–14	17	12	71	0.61 (0.38–0.99)		0.42 (0.19–0.89)	
15–259	19	11	58	0.37 (0.14–0.98)		0.17 (0.04–0.80)	
>259	18	11	61	0.23 (0.05–0.98)	0.046	0.07 (0.01–0.72)	0.025
IL12							
≤−0.5	18	15	83	1.00		1.00	
−0.4–0.4	18	12	67	0.63 (0.39–1.02)		0.43 (0.21–0.92)	
0.5–1.2	18	15	83	0.40 (0.15–1.05)		0.19 (0.04–0.84)	
>1.2	19	9	47	0.25 (0.06–1.07)	0.061	0.08 (0.01–0.77)	0.029

*n* = number of; g/L = grams per Litre; IgM = immunoglobulin M; CCL19 = C-C motif chemokine ligand 19; CD70 = cluster of differentiation 70; CXCL13 = C-X-C motif chemokine ligand 13; IL13 = interleukin 13; GZMB = Granzyme B; IL12 = interleukin 12.

## Data Availability

The data presented in this study are available on request from the corresponding author. The data are not publicly available due to privacy reasons.

## Supplementary Materials

The following supporting information can be downloaded at https://www.mdpi.com/article/10.3390/vaccines12030303/s1: Table S1: Protein profile from PBMCs stimulated with influenza vaccine, adjusted for background concentration and analysed with the OLINK Oncology II panel via a proximity extension assay. Median values, quartiles (Q1–Q3) and *p*-values below 0.10 are shown. NPX values are on a linear scale; Table S2: Protein biomarker characteristics of participants with immunosuppressive medication and vaccine breakthrough.
