# Supplementary material for: Cell-Mediated Proteomics, and Serological and Mucosal Humoral Immune Responses after Seasonal Influenza Immunization: Characterization of Serological Responders and Non-Responders
Source: Vaccines (Basel). 2024 Mar 14;12(3):303. doi: 10.3390/vaccines12030303 (PMC10975048; doi:10.3390/vaccines12030303)
Supplement: Supplementary file 1 [file vaccines-12-00303-s001.zip › vaccines-2880426-Table S1.pdf]

Table S1. Proteins profile from PBMC stimulated with influenza vaccine, adjusted for background concentration analyzed with the Olink Oncology II panel analysed by Proximity Extension Assay. Median values, quartiles (Q1-Q3), and P-values below 0.10 are shown. NPX values are in a linear scale.

				Inclusion			One month post vaccination			Six months post vaccination		
**Assay**	**Uniprot ID**	**LOD**	**<LOD (%)**	**Responders**	**Nonresponders**	**p**	**Responders**	**Nonresponders**	**p**	**Responders**	**Nonresponders**	**p**
N				51	21		51	22		47	21	
ADA	P00813	2,30	0%	28.5 (-11.7-93.1)	26.1 (-29.7-122.8)		8.8 (-30.6-80.8)	17.6 (-2.4-85.7)		29.1 (-19.9-71.6)	23.5 (-10.1-62.8)	
ADGRG1	Q9Y653	3,51	100%	0.5 (-0.7-1.1)	0.2 (-0.7-1.2)		0.6 (-0.7-1.6)	0.5 (-0.5-1.5)		0.0 (-1.1-1.9)	-0.2 (-1.2-0.4)	
ANGPT1	Q15389	1,40	0%	-1.5 (-4.5-2.4)	0.1 (-4.4-1.8)		-0.3 (-3.9-5.9)	-0.1 (-7.8-10.9)		1.1 (-2.6-12.9)	0.8 (-3.1-6.5)	
ANGPT2	O15123	2,48	100%	0.0 (-0.3-0.4)	0.1 (-0.4-0.4)		-0.1 (-0.5-0.6)	0.2 (-0.6-0.6)		0.1 (-0.6-0.5)	0.1 (-0.5-0.6)	
ARG1	P05089	3,63	96%	0.2 (-0.3-0.6)	0.3 (-0.2-0.4)		0.1 (-0.1-0.8)	0.3 (-0.2-0.6)		0.3 (-0.1-0.7)	0.1 (-0.2-0.5)	
CAIX	Q16790	1,82	100%	0.1 (-0.2-0.4)	0.0 (-0.5-0.2)		0.2 (-0.2-0.4)	0.2 (-0.3-0.6)		0.0 (-0.4-0.4)	-0.3 (-0.5-0.5)	
CASP-8	Q14790	3,12	0%	-4.7 (-49.4-47.0)	-3.7 (-31.9-50.8)		10.6 (-29.1-69.4)	-3.2 (-57.0-61.0)		12.0 (-23.4-85.9)	-0.8 (-27.2-66.3)	
CCL17	Q92583	1,66	0%	2.0 (-2.3-5.4)	1.0 (-3.6-7.8)		2.7 (-0.3-12.0)	4.6 (-0.5-21.4)		3.5 (-2.7-17.3)	2.6 (-3.9-10.9)	
CCL19	Q99731	3,50	49%	0.5 (-1.3-1.6)	1.4 (-1.1-5.6)		1.2 (-0.4-3.2)	3.5 (-0.3-11.4)	0,041	0.7 (-1.4-3.1)	1.1 (0.0-4.6)	
CCL20	P78556	3,24	33%	0.5 (-0.9-3.3)	1.4 (-0.9-3.7)		2.1 (0.0-6.8)	3.5 (-1.3-13.1)		4.9 (0.2-15.5)	1.8 (-0.8-12.5)	
CCL23	P55773	1,40	33%	0.0 (-0.1-0.3)	0.2 (-0.1-0.4)		0.2 (-0.1-0.4)	0.4 (0.0-0.8)		0.3 (0.0-0.5)	0.3 (0.0-0.6)	
CCL3	P10147	2,38	0%	42 (-6-137)	185 (-55-716)		137 (18-613)	299 (32-965)		145 (32-400)	81 (11-418)	
CCL4	P13236	2,88	0%	20 (-9-60)	59 (-23-246)		50 (2-163)	48 (11-367)		62 (14-175)	45 (6-326)	
CD244	Q9BZW8	3,62	0%	6.6 (-2.2-24.1)	8.0 (-5.6-24.3)		5.8 (-0.1-19.8)	8.2 (1.4-24.1)		18.0 (1.0-42.1)	16.7 (4.1-26.2)	
CD27	P26842	1,53	0%	8.5 (1.0-20.8)	10.2 (0.4-31.7)		8.4 (3.2-23.6)	9.9 (2.5-23.1)		11.1 (5.2-30.5)	11.6 (3.3-28.0)	
CD28	P10747	3,63	99%	0.1 (-0.9-1.5)	0.0 (-2.3-1.9)		0.4 (-1.1-1.9)	0.8 (-0.7-1.6)		0.3 (-1.9-2.0)	-0.6 (-1.4-1.0)	
CD4	P01730	3,34	0%	-0.2 (-6.4-8.0)	0.0 (-7.7-4.1)		0.3 (-5.2-5.9)	0.3 (-5.6-7.3)		3.7 (-4.2-11.9)	1.7 (-0.5-4.4)	
CD40	P25942	2,98	0%	-19 (-138-148)	-15 (-205-73)		-13 (-123-149)	-15 (-102-130)		68 (-61-358)	53 (-89-95)	
CD40-L	P29965	4,07	40%	-0.4 (-2.6-3.8)	0.1 (-2.7-2.1)		1.9 (-1.2-4.5)	2.9 (0.2-4.8)		1.6 (-1.2-6.8)	0.0 (-1.2-2.7)	
CD5	P06127	2,11	0%	1 (-102-222)	-12 (-282-157)		-1 (-71-121)	34 (-83-129)		53 (-19-295)	63 (-35-153)	
CD70	P32970	2,10	34%	0.1 (-0.3-0.8)	0.6 (-0.5-1.9)		0.5 (-0.1-2.0)	1.5 (-0.1-4.0)	0,070	0.7 (-0.2-1.8)	0.5 (0.0-1.9)	
CD83	Q01151	1,45	0%	1.2 (-0.2-2.1)	1.5 (-0.5-3.0)		0.9 (0.1-2.4)	1.6 (0.4-3.0)		1.2 (0.1-3.4)	1.0 (0.4-2.9)	
CD8A	P01732	2,90	0%	-3.6 (-57.2-26.7)	-12.3 (-36.9-14.0)		-3.6 (-20.4-16.8)	-0.4 (-33.5-51.7)		-3.0 (-23.1-86.6)	2.5 (-10.3-22.8)	
CRTAM	O95727	2,84	84%	0.1 (-0.3-1.1)	0.1 (-1.0-1.3)		0.2 (-0.6-1.2)	0.7 (-0.4-2.0)		0.0 (-0.6-1.4)	0.1 (-0.7-0.7)	
CSF-1	P09603	3,12	0%	21.6 (4.3-63.7)	44.8 (15.9-169.5)		34.8 (9.3-149.8)	54.2 (17.7-305.5)		53.7 (16.8-158.5)	56.3 (13.3-236.1)	
CX3CL1	P78423	1,71	95%	-0.1 (-0.4-0.2)	-0.1 (-0.3-0.4)		0.1 (-0.2-0.3)	0.1 (-0.1-0.5)		0.1 (-0.2-0.5)	0.2 (-0.3-0.4)	
CXCL1	P09341	3,27	0%	12 (-71-439)	144 (-132-751)		52 (-116-1595)	198 (-12-1646)		509 (-29-1462)	337 (-45-1014)	
CXCL10	P02778	3,42	0%	77 (5-540)	323 (67-1723)		389 (40-1589)	1072 (57-16251)		590 (153-1533)	971 (317-1874)	
CXCL11	O14625	1,99	0%	0.0 (-2.4-4.0)	3.9 (-2.6-28.7)		1.5 (-0.8-15.8)	8.5 (0.7-63.4)		3.6 (-0.7-10.1)	3.5 (1.6-15.8)	
CXCL12	P48061	2,56	100%	0.0 (-0.3-0.4)	0.0 (-0.4-0.9)		0.1 (-0.4-0.6)	0.0 (-0.3-0.6)		0.0 (-0.6-0.6)	0.1 (-0.2-0.3)	
CXCL13	O43927	3,31	50%	-0.4 (-1.4-0.9)	-0.6 (-3.0-2.1)		0.6 (-1.5-2.7)	-1.1 (-5.2-2.5)	0,068	0.8 (-1.0-2.9)	0.3 (-2.2-1.5)	
CXCL5	P42830	3,48	0%	-4 (-837-850)	545 (-94-1077)		45 (-840-1101)	-355 (-909-1126)		54 (-1083-856)	-53 (-658-629)	
CXCL9	Q07325	2,73	1%	19 (4-96)	71 (14-242)		54 (14-187)	94 (21-621)		76 (20-167)	101 (34-177)	
DCN	P07585	2,74	100%	0.0 (-0.2-0.2)	0.1 (-0.2-0.3)		0.1 (-0.1-0.4)	0.1 (-0.3-0.6)		0.1 (-0.3-0.4)	0.1 (-0.1-0.2)	
EGF	P01133	2,02	0%	4.5 (-3.9-22.4)	5.1 (-0.5-16.7)		7.4 (-3.6-44.4)	10.0 (-8.0-35.1)		15.3 (1.9-58.1)	19.1 (-1.9-36.2)	
FASLG	P48023	2,25	0%	2.4 (-2.6-9.3)	3.3 (-6.3-10.8)		2.5 (-1.5-11.9)	7.8 (1.7-16.1)		3.0 (-2.1-11.9)	5.7 (-0.2-8.8)	
FGF2	P09038	2,05	85%	0.1 (-0.2-0.7)	0.3 (-0.4-0.9)		0.4 (-0.2-0.9)	0.5 (-0.2-0.8)		0.3 (-0.3-0.7)	0.3 (-0.2-1.1)	
Gal-1	P09382	3,53	0%	1.6 (-13.1-15.4)	5.7 (-6.4-21.1)		6.2 (-9.1-21.2)	10.3 (-7.3-24.5)		8.0 (-5.7-29.9)	12.5 (-0.9-18.2)	
Gal-9	O00182	2,57	0%	27.0 (-21.4-95.7)	31.4 (11.0-66.8)		27.1 (-10.7-135.1)	49.2 (-2.0-118.4)		59.7 (4.2-178.1)	44.9 (-10.3-100.4)	

GZMA	P12544	1,88	0%	53 (-466-1174)	347 (-1093-1868)			-25 (-915-1556)	306 (-435-1333)			585 (-239-2749)	911 (203-2012)	
GZMB	P10144	2,80	0%	0 (-109-186)	71 (-249-294)			38 (-108-385)	147 (74-684)	0,066		149 (-99-439)	188 (33-339)	
GZMH	P20718	3,05	0%	-22 (-286-264)	77 (-346-318)			-65 (-337-403)	135 (-132-443)			288 (-231-960)	428 (52-622)	
HGF	P14210	2,08	0%	0.0 (-1.6-2.8)	-1.2 (-4.3-2.7)			-0.3 (-3.0-3.8)	0.5 (-2.5-4.0)			1.3 (-0.9-7.8)	0.9 (0.0-2.6)	
HO-1	P09601	3,50	0%	29.0 (-61.8-158.1)	2.1 (-83.9-52.1)			25.6 (-115.5-238.2)	1.2 (-97.0-94.9)			70.8 (-83.6-266.5)	18.3 (-32.9-54.6)	
ICOSLG	O75144	2,55	0%	0.3 (-0.6-1.7)	0.4 (-0.1-1.6)			0.7 (-0.5-2.0)	0.4 (-0.7-2.0)			1.1 (-0.3-1.8)	0.8 (0.0-1.9)	
IFN-gamma	P01579	4,37	0%	2319 (446-8378)	2810 (416-16622)			3406 (757-11589)	4545 (967-66957)			4388 (1843-10021)	5055 (1297-13150)	
IL-1 alpha	P01583	5,05	100%	0.1 (-0.1-0.2)	0.1 (-0.5-0.2)			0.0 (-0.1-0.4)	0.1 (-0.2-0.5)			0.1 (-0.1-0.4)	0.3 (0.0-1.3)	
IL10	P22301	3,00	67%	0.5 (-0.4-1.6)	0.6 (-1.0-1.7)			1.8 (0.5-4.0)	3.0 (0.9-8.9)			1.8 (0.4-3.5)	1.6 (0.5-3.1)	
IL12	P29459,P29460	2,89	91%	0.3 (-0.4-1.1)	0.5 (-1.0-1.4)			0.8 (-0.4-1.7)	1.8 (0.6-3.6)	0,035		0.8 (-0.4-1.7)	0.4 (0.0-1.7)	
IL12RB1	P42701	3,29	98%	0.1 (-0.5-1.0)	-0.1 (-1.1-0.9)			0.1 (-0.7-1.7)	1.1 (-0.6-1.8)			0.5 (-0.7-1.4)	0.4 (-0.7-0.9)	
IL13	P35225	3,22	98%	0.0 (-0.8-0.8)	-0.6 (-1.0-0.3)			0.3 (-0.7-1.9)	1.0 (0.3-3.3)	0,024		0.2 (-0.5-1.1)	0.6 (-0.2-1.2)	
IL15	P40933	4,05	91%	0.9 (-0.2-2.0)	1.7 (-2.0-3.0)			1.4 (0.2-5.0)	2.3 (0.1-9.8)			1.6 (0.2-3.8)	2.2 (0.7-3.4)	
IL18	Q14116	2,16	0%	0.6 (-1.3-5.3)	-0.7 (-1.7-3.2)			-0.2 (-2.4-3.8)	1.4 (-1.7-4.0)			1.2 (0.0-6.1)	2.8 (-0.5-4.8)	
IL2	P60568	2,81	69%	2.7 (0.5-8.5)	3.3 (1.2-26.3)			4.8 (1.8-23.4)	4.8 (1.7-145.9)			5.9 (2.5-17.6)	5.0 (2.0-11.2)	
IL33	O95760	2,28	99%	0.1 (-0.4-0.3)	-0.1 (-0.4-0.5)			0.0 (-0.4-0.7)	0.2 (-0.4-0.7)			0.3 (-0.5-0.7)	0.0 (-0.5-0.4)	
IL4	P05112	2,28	98%	0.2 (-0.2-0.7)	0.4 (-0.4-0.8)			0.1 (-0.4-1.0)	0.3 (-0.4-1.2)			0.3 (-0.4-0.9)	0.4 (-0.1-1.0)	
IL5	P05113	2,90	98%	0.4 (-0.3-1.1)	-0.3 (-0.9-1.4)			0.1 (-1.0-1.0)	0.7 (-0.5-1.8)			0.4 (-1.1-1.1)	-0.2 (-0.5-0.6)	
IL6	P05231	3,74	1%	3.3 (-1.5-12.9)	9.6 (-3.2-23.4)			15.4 (5.2-36.6)	10.4 (2.1-63.7)			17.4 (1.0-33.0)	8.0 (4.1-56.2)	
IL7	P13232	1,92	23%	0.2 (-0.4-0.6)	-0.1 (-0.8-0.4)			0.1 (-0.3-0.7)	0.3 (-0.1-0.6)			0.1 (-0.3-0.7)	0.4 (-0.5-1.1)	
IL8	P10145	3,50	0%	299 (-1032-769)	-173 (-929-3371)			-151 (-1705-760)	-473 (-1244-871)			-937 (-1826-13)	182 (-1637-1062)	
KIR3DL1	P43629	3,22	89%	0.3 (-0.5-1.0)	0.0 (-2.0-1.0)			0.2 (-1.0-1.2)	0.1 (-0.5-1.6)			0.3 (-0.8-1.4)	0.4 (-0.9-1.1)	
KLRD1	Q13241	2,14	1%	0.8 (-0.6-2.2)	0.6 (-0.8-3.2)			0.6 (-0.1-2.8)	0.9 (0.2-4.1)			1.7 (-0.2-3.1)	1.3 (0.8-2.6)	
LAG3	P18627	3,18	97%	0.0 (-0.4-0.4)	0.4 (-0.7-0.9)			0.5 (-0.5-1.1)	0.7 (-0.1-1.5)			0.2 (-0.5-0.8)	0.3 (-0.8-1.1)	
LAMP3	Q9UQV4	2,62	98%	0.0 (-0.5-0.6)	-0.1 (-0.9-0.5)			0.0 (-0.6-0.6)	0.2 (-0.9-0.7)			0.0 (-0.5-0.9)	0.2 (-0.9-0.7)	
LAP TGF-beta-1	P01137	1,86	0%	5.6 (-6.1-42.8)	19.2 (0.8-52.0)			11.3 (-0.2-34.5)	8.1 (-20.3-73.1)			17.6 (-1.4-68.0)	27.9 (-1.3-42.7)	
MCP-1	P13500	2,80	0%	774 (64-2616)	1669 (118-6692)			2487 (235-8691)	2632 (302-9787)			3283 (848-7927)	3003 (500-6217)	
MCP-2	P80075	2,67	0%	27 (2-148)	137 (12-1006)	0,074		131 (11-745)	424 (12-1197)			180 (76-566)	466 (54-723)	
MCP-3	P80098	3,24	0%	22 (5-132)	71 (2-664)			98 (10-340)	196 (9-1102)			222 (48-530)	264 (50-815)	
MCP-4	Q99616	2,04	0%	13 (1-51)	35 (1-196)			47 (5-184)	102 (13-306)			62 (28-178)	94 (23-202)	
MIC-A/B	Q29983,Q29980	2,69	92%	0.1 (-0.4-0.6)	0.1 (-0.7-1.0)			0.2 (-0.5-1.0)	0.1 (-0.2-1.3)			0.2 (-0.2-0.6)	0.2 (-0.1-0.8)	
MMP12	P39900	2,33	86%	0.1 (-0.4-0.5)	0.3 (-0.3-1.1)			0.4 (-0.2-1.0)	0.5 (0.3-5.3)			0.4 (-0.2-0.7)	0.1 (-0.1-0.7)	
MMP7	P09237	2,71	0%	5.2 (1.2-14.2)	11.9 (-3.1-38.1)			13.2 (2.6-47.8)	9.0 (3.7-81.4)			15.3 (3.9-35.2)	17.4 (5.1-48.9)	
MUC-16	Q8WXI7	2,30	100%	0.0 (-0.4-0.4)	0.0 (-0.7-0.5)			0.0 (-0.2-0.5)	0.0 (-0.4-0.8)			0.1 (-0.6-0.6)	0.1 (-0.4-0.5)	
NCR1	O76036	2,77	81%	0.4 (-1.0-1.0)	0.0 (-1.0-1.0)			0.2 (-0.4-1.3)	0.4 (-0.1-1.4)			0.7 (-0.7-1.2)	0.7 (0.0-1.0)	
NOS3	P29474	3,88	100%	0.2 (-1.1-1.6)	-0.9 (-2.3-1.2)			0.0 (-1.2-1.3)	0.5 (-0.8-2.2)			-0.1 (-1.7-1.6)	-0.2 (-1.7-1.1)	
PDCD1	Q15116	2,57	56%	0.3 (-1.0-1.6)	0.5 (-1.6-1.6)			0.6 (-0.2-1.1)	0.8 (-0.4-2.3)			0.8 (-0.8-1.7)	0.2 (-0.1-1.3)	
PDGF subunit B	P01127	1,25	0%	3.8 (-8.8-22.8)	0.4 (-16.9-12.7)			5.3 (-15.5-41.3)	6.6 (-14.2-33.9)			17.3 (-0.3-78.1)	18.8 (-4.8-37.5)	
PD-L1	Q9NZQ7	2,97	3%	1.5 (-1.0-5.1)	2.0 (0.7-9.6)			3.8 (-0.2-9.2)	4.7 (2.4-16.8)			4.0 (-0.3-14.3)	3.4 (2.0-10.5)	
PD-L2	Q9BQ51	2,87	99%	0.1 (-0.4-0.6)	0.0 (-0.7-0.6)			0.1 (-0.5-0.6)	0.2 (-0.2-0.8)			0.1 (-0.5-0.7)	0.1 (-0.4-0.6)	
PGF	P49763	2,97	9%	0.2 (-0.7-1.3)	0.6 (-1.2-1.4)			0.5 (-0.7-1.7)	0.2 (-1.0-1.5)			0.7 (-0.5-1.5)	0.7 (-0.6-1.3)	
PTN	P21246	3,19	100%	0.1 (-0.8-1.0)	0.3 (-0.8-0.9)			-0.1 (-1.3-1.1)	0.5 (-0.6-1.1)			-0.1 (-1.3-1.4)	-0.1 (-0.9-0.6)	
TIE2	Q02763	2,63	95%	0.0 (-0.5-0.5)	0.3 (-0.7-0.8)			0.1 (-0.6-1.0)	0.4 (-0.7-1.1)			0.1 (-1.1-1.0)	0.1 (-0.7-0.7)	
TNF	P01375	3,00	0%	11.0 (1.0-33.8)	22.2 (5.9-154.6)			19.7 (4.6-53.3)	20.9 (4.7-221.6)			17.7 (7.4-58.6)	13.6 (6.0-80.6)	

TNFRSF12A	Q9NP84	2,28	100%	0.0 (-0.4-0.5)	0.1 (-0.7-0.4)			0.1 (-0.3-0.4)	0.3 (-0.2-0.6)			0.2 (-0.4-0.5)	-0.2 (-0.5-0.4)	
TNFRSF21	O75509	-0,02	0%	0.1 (-0.1-0.3)	0.2 (0.0-0.3)			0.1 (0.0-0.3)	0.0 (-0.2-0.2)			0.2 (0.0-0.3)	0.2 (0.0-0.3)	
TNFRSF4	P43489	4,90	87%	0.8 (-2.1-5.4)	1.0 (-5.3-6.5)			1.9 (-2.3-5.3)	2.5 (-3.4-7.7)			2.5 (-1.2-7.0)	1.5 (-1.3-5.6)	
TNFRSF9	Q07011	1,08	0%	1.9 (0.2-7.0)	2.8 (0.5-6.9)			1.8 (0.2-7.8)	4.5 (-0.2-7.6)			3.6 (0.9-9.3)	3.8 (2.1-9.5)	
TNFSF14	O43557	3,51	0%	8.4 (-2.5-22.7)	10.8 (-0.1-31.3)			8.8 (-0.6-30.4)	15.6 (4.2-25.7)			15.8 (0.8-41.7)	11.5 (5.8-32.5)	
TRAIL	P50591	2,85	44%	0.6 (-0.5-1.3)	0.4 (-1.5-1.9)			1.4 (-0.1-2.3)	2.3 (-0.5-4.8)			0.8 (-0.3-2.5)	0.9 (0.1-1.8)	
TWEAK	O43508	1,20	0%	0.0 (-1.9-1.4)	-0.6 (-4.3-1.6)			-0.1 (-1.6-2.0)	1.0 (-0.3-2.7)			1.7 (-0.9-4.1)	1.0 (0.0-1.8)	
VEGFA	P15692	3,16	0%	3.2 (-2.1-12.0)	4.2 (-13.9-15.4)			6.1 (-4.1-12.6)	1.9 (-4.3-8.2)			7.9 (0.4-45.6)	6.0 (0.7-22.6)	
VEGFR-2	P35968	2,86	75%	0.3 (-0.8-1.1)	0.3 (-0.8-1.5)			0.0 (-0.8-1.1)	0.3 (-0.6-1.1)			0.2 (-1.1-1.4)	-0.1 (-1.1-0.6)	
