# Supplementary material for: Cell-Mediated Proteomics, and Serological and Mucosal Humoral Immune Responses after Seasonal Influenza Immunization: Characterization of Serological Responders and Non-Responders
Source: Vaccines (Basel). 2024 Mar 14;12(3):303. doi: 10.3390/vaccines12030303 (PMC10975048; doi:10.3390/vaccines12030303)
Supplement: Supplementary file 1 [file vaccines-12-00303-s001.zip › vaccines-2880426-Table_S2.pdf]

Table S2. Protein biomarkers characteristics of participant with immunosuppressive medication and vaccine breakthrough

Parameter	Median (responders)	Median (non-responders)	Immunosuppressive medication	Vaccine breakthrough
**IgM (g/L)**	0.71	0.9	0.68	0.64
**CCL19**	1.17	3.47	-0.16	1.33
**CD70**	0.51	1.52	0.26	-0.009
**CXCL13**	0.62	-1.09	2.32	2.66
**IL13**	0.31	1.02	0.63	-0.25
**GZMB**	38.19	146.5	-107.49	757.59
**IL12**	0.76	1.81	1.21	-0.997

The parameters displayed in the table are the once that were included in the univariate and multivariate logistic regression for further characterisation of the responders and non-responders. The unit of the protein biomarkers except for total IgM are linear NPX- values.
